# Life with a Gastric Band. Long-Term Outcomes of Laparoscopic Adjustable Gastric Banding—a Retrospective Study

**DOI:** 10.1007/s11695-016-2435-2

**Published:** 2016-10-27

**Authors:** Piotr K. Kowalewski, Robert Olszewski, Andrzej Kwiatkowski, Natalia Gałązka-Świderek, Krzysztof Cichoń, Krzysztof Paśnik

**Affiliations:** 1 Department of General Surgery, Military Institute of Medicine, Szaserów 128, 04-141 Warsaw, Poland; 2 Department of Cardiology, Military Institute of Medicine, Warsaw, Poland; 3 Department of Internal Medicine, Regional Hospital, Łęczna, Poland

**Keywords:** Laparoscopy, Bariatric surgery, LAGB, Gastric band, Long term follow-up

## Abstract

**Background:**

Laparoscopic adjustable gastric banding (LAGB) is the third most popular bariatric procedure worldwide. Various authors present ambivalent long-term follow up results.

**Methods:**

We revised records of the patients who underwent LAGB between 2003 and 2006 along with history of additional check-ins. Patients with outdated details were tracked with the national health insurance database and social media (Facebook). An online survey was sent. The patients who did not have their band removed were included in this study. We calculated the percent total weight loss (%TWL) and percent excess weight loss (%EWL), along with changes in body mass index (ΔBMI). Satisfactory weight loss was set at >50% EWL (for BMI = 25 kg/m^2^). Since eight patients gained weight, we decided to include negative values of %TWL, %EWL, and ΔBMI.

**Results:**

One hundred seven patients underwent LAGB from 2003 to 2006. The mean follow-up time was 11.2 (±1.2) years. Eleven percent of patients were lost to follow up (*n* = 12). There was one perioperative death. Fifty-four of the patients (*n* = 57) had their band removed. Thirty-seven patients still have the band (39%) and were included in the study. The mean %EWL was 27% (−56–112%) and %TWL was 11% (−19–53%). Twelve patients achieved %EWL > 50% (32%). Thiry-two patients still suffer from obesity, with BMI over 30 kg/m^2^. Eight patients (22%) gained additional weight. Patients with %EWL > 50% suffered less from gastroesophageal reflux disease symptoms than those with EWL < 50% (*p* < 0.05).

**Conclusions:**

Out of 107 cases, only 11.2% of patients with gastric band (*n* = 12) achieved satisfactory %EWL. Twenty–two percent of patients regained their weight or even exceeded it. Overall results suggest that LAGB is not an effective bariatric procedure in long term observation.

## Introduction

Adjustable gastric banding was first introduced as a bariatric procedure in the mid-80s [1]. Its overall simplicity, and the development of laparoscopy allowed surgeons to implement a minimally invasive technique, which, by this day is known as laparoscopic adjustable gastric banding (LAGB) [2]. It is the third most popular bariatric surgery, representing 10% of all the procedures in 2013 [3]. Interestingly, in 2008 LAGB was the most commonly performed procedure for patients suffering from obesity, with 42.3% of all bariatric surgeries [4]. Bearing in mind this decrease, still over 40,000 patients undergo this operation worldwide every year, according to the 2013 survey. The overall change in popularity may be attributed to doubts regarding its effectiveness in the long-term research [5]. Yet, a number of large, prospective studies acknowledge LAGB as a successful bariatric procedure [6]. In our study, we would like to focus on the long-term history of patients who underwent LAGB and still live with a gastric band.

## Aim

To evaluate long-term clinical outcomes of laparoscopic adjustable gastric banding (LAGB) regarding weight loss, physical activity and complaints of gastroesophageal reflux disease (GERD) in patients are living with the gastric band.

## Materials and Methods

Our institution database was revised for the record of patients who underwent LAGB between 2003 and 2006. We have gathered the data on their weight, body mass index (BMI), and co-morbidities. The history of additional check-ins was revised. The patients who did not fulfill their check-ins and whose personal data was outdated were tracked using the national health insurance database, or found using social media (Facebook and LinkedIn) and its support groups. These patients received and filled out an online questionnaire regarding their current weight, medical history, complaints of GERD, physical activity habits—expressed by regular, over 30-min exercise routine, and sedentary behavior, assessed by hours spent siting down on a daily basis. The patients who did not have their band removed were included in this study. To measure the effectiveness of the procedure, the percentage total weight loss (%TWL), percentage excess weight loss (%EWL), and change in body mass index (ΔBMI) were calculated.

We calculated the excess weight (EW) from the weight before surgery subtracted by ideal body weight (IBW) for BMI of 25 kg/m^2^. Satisfactory weight loss after the surgery was defined by the EWL greater than 50%.

We performed the statistical analysis using “Statistica” software (StatSoft). Normality of the data was tested with Shapiro-Wilk test. Continuous variables were compared with the student *t* test. Categorical variables were compared using the chi^2^ test. Statistical significance was set at *p* < 0.05.

Since eight patients gained weight, we decided to include negative values of %TWL, %EWL, and ΔBMI.

## Results

**Fig. 1:**
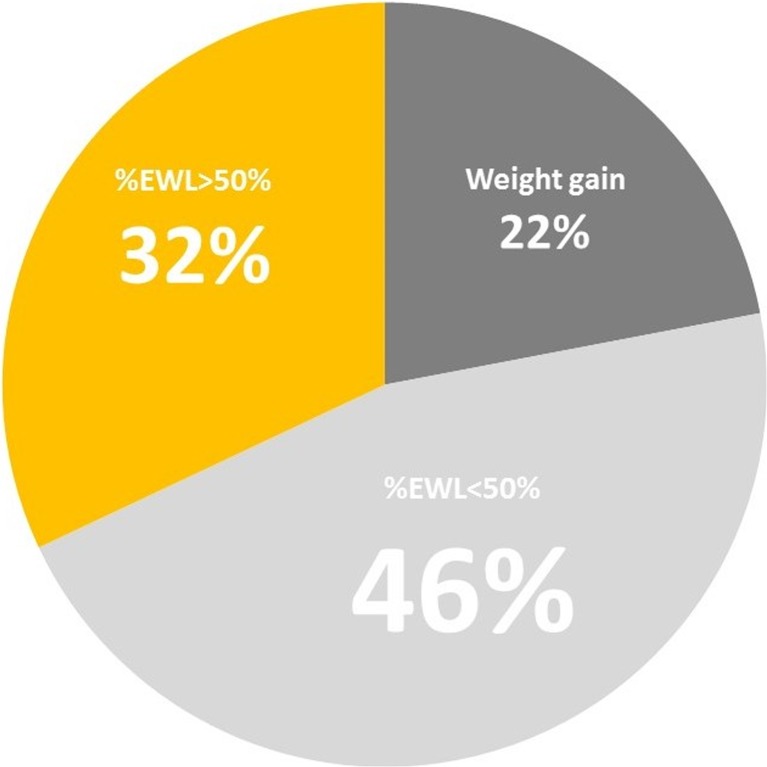
Long term outcomes of LAGB

One hundred seven patients underwent LAGB between 2003 and 2006 in our institution. There was one perioperative death (0.09%). Eleven percent of patients were lost to follow up **(** ***n*** **=** 12). Fifty-four percent of patients (*n* = 57) had their band removed. Out of the remaining group, 39% of patients (*n* = 37) still live with a functioning gastric band and were included in this study. The mean follow-up time was 11.2 (±1.2) years. There were 78% female (*n* = 29) and 22% male (*n* = 8) patients. The mean patients’ age was 37 years (ranging from 20 to 63) and mean BMI before the surgery was 42.3 (±4.3) (Table 1). Several complications led to the band removal. The main causes were the following: weight gain (40%), pouch slippage (25%), port complications, such as infection and dislocation (21%), pouch dilatation (16%), and band erosion (5%) (Table 2)**.** The long-term outcomes of LAGB in the study group are presented in Table 3 and Fig. 1. The mean excess weight loss was 27% (−56–112%) and total weight loss was 11% (−19%–53%). Thirty-two percent of patients achieved %EWL > 50% (*n* = 12). 86% of patients (*n* = 32) still suffer from obesity, with BMI over 30 kg/m^2^. It should be noted that eight patients gained additional weight (22%), exceeding their body mass from before the surgery. Interestingly, mean maximum %EWL recorded in the follow-up reached 69%. Over half (57%) of the patients achieved it in the first year after the surgery.

**Table 1:** Demographic data

	Value	Percent (SD) [range]
Gender (female/male)	29/8	78%/22%
Age	37	[20–63]
Mean weight (kg)	120.7	(±17.0) [90–150]
Mean BMI (kg/m^2^ )	42.3	(±4.3) [33.7–52.1]
Mean excess weight (kg)	49.3	(±12.7) [24.4–76.0]

**Table 2:** Causes of revisional surgery after LAGB

Causes	Number	Percent
Weight gain	23	40
Slippage	14	25
Pouch dilatation and reflux	9	16
Band erosion	3	5
Port problem (infection, dislocation)	12	21

**Table 3:** Long term outcomes of LAGB

	Value	Percent (SD) [range]
Time of follow-up (years)	11.2	(±1.1)
%EWL	27%	(±38%) [−56–112%]
%TWL	11%	(±15%) [−19–53%]
ΔBMI	4.71	(±6.65) [−7.3–25.3]
Number of patients with %EWL > 50%	12	33%
Number of patients with weight gain	8	22%

Regarding physical activity and habits, 15 patients (41%) exercised regularly for at least 30 min daily. Mean sedentary time in all patients was 7 h per day. Therefore, the patients were divided into two groups according to the achieved %EWL over and under 50%, physical activity, and gained weight. These groups were then compared regarding different parameters. There was a statistically significant difference in GERD symptoms reported between patients with %EWL > 50% (group 1) and %EWL < 50% (group 2). Only 5 (42%) patients from group 1 suffered from GERD, compared to 19 (79%) from group 2 (*p* < 0.05). Other differences were not statistically significant (Table 4).

**Table 4:** Difference between patients with %EWL > 50% and %EWL < 50%

	Group 1 (EWL > 50%) *n* = 12	Group 2 (EWL < 50%) *n* = 25	*p* value
GERD symptoms	5 (42%)	20 (80%)	<0.05^a^
Sedentary hours (mean)	5.8 (±3)	7.6 (±2.6)	*p* = 0.069^b^
Physically active	6 (50%)	9 (36%)	*p* = 0.41^a^

^a^Chi^2^ test

^b^ *t* student test

## Discussion

According to WHO, obesity affected 600 million people worldwide in 2014. Thirteen percent of the world population was suffering from obesity. These numbers doubled since 1980. Over the years, new methods of treatment became available to patients. Current research supports bariatric surgery as the most effective one*.* [7, 8]. Out of all the variety of existing procedures, LAGB is the third most popular in the world, according to the survey from 2013. Ten percent of the bariatric patients, almost 40,000 people worldwide, undergo this procedure every year [3]. In Poland, LAGB is preceded by laparoscopic sleeve gastrectomy (LSG), laparoscopic Roux-en-Y gastric bypass (LRYGB), and mini gastric bypass (MGB). Adjustable band accounts for 7.6% of bariatric procedures [9]. There are several qualities that make LAGB such a popular procedure. It is fairly uncomplicated in the hands of an experienced surgeon. It has good short-term effects, and is mostly reversible. Even in our study, 69 % of the patients achieved satisfying %EWL over 50% at some point after the surgery. Yet when it comes to long-term results, the outcomes are not as favorable. Although O’Brien et al. report a stunning 47% excess weight loss in a 15-year follow-up, yet over 50% of patients had their band removed, with or without additional bariatric surgery, such as LRYGB or LSG [6]. Other authors do not present such favorable long-term outcomes of LAGB. Aarts et al. report that only 22% of all LAGB patients maintain a functioning band with good results. Fifty-three percent of the patients underwent a revisional procedure and/or band removal [10]. Sutter et al. underlines linear progression of major complications and even states in the conclusion that LAGB should not be considered a procedure of choice for treating obesity [5]. Himpens et al. also reaches the conclusion that LAGB long term effects are poor due to a high reoperation rate [11]. When it comes to co-morbidities, its reduction in short-term follow-up is not maintained in the long term [10]. Out of many studied co-morbidities, only glucose tolerance improves and maintains its improvement in long term observation [12]. GERD and its symptoms are also frequently mentioned regarding bariatric surgery. Naik et al. state that adjustable gastric banding worsens GERD or even forms de novo cases in previously asymptomatic patients [13]. Greater amount of GERD and dysphagia symptoms in patients with %EWL lower than 50% in our study may suggest dietary inaccuracy in this particular group. Overall, bariatric surgery tends to improve patients’ physical activity [14]. Yet, in our observation, only 41% of patients remain physically active, with average daily sedentary time as long as 7 h.

## Conclusions

Only 39% of patients still maintain a functioning band after over 10 years. The rest of the patients had their band removed due to various complications. Out of all of the patients, only 11.2% of them achieved satisfactory %EWL over 50%, which means that the band fail rate reached 88.8%. Twenty-two percent of patients from the study group regained their weight or even exceeded it. Over half of the patients suffer from dysphagia, more often when weight loss is not satisfactory, which suggests that these patients do not comply to dietary guidelines. Overall results suggest that LAGB is not an effective bariatric procedure in long term observation.
